# An unusual cause of visual loss after Herpes zoster ophthalmicus: a case report

**DOI:** 10.1186/1757-1626-3-17

**Published:** 2010-01-12

**Authors:** Jayne E Camuglia, Jacqueline E Beltz, Kavita Khurana, Anthony JH Hall

**Affiliations:** 1 Department of Ophthalmology, The Alfred Hospital, Commercial Road, Prahran, Melbourne, Victoria 3004, Australia

## Abstract

**Introduction:**

The vascular complications of herpes zoster are well recognised, however, there are few reports of central retinal artery occlusion. Central retinal artery occlusion following herpes zoster ophthalmicus is poorly recognised. This is likely due to the difficulties in obtaining tissue for histopathology to establish causality. We report a case of central retinal artery occlusion and complete internal carotid artery occlusion following herpes zoster ophthalmicus.

**Case presentation:**

A 44 year old Caucasian female presented with sudden painless loss of vision in her right eye on a background of chronic lymphocytic leukaemia and right sided herpes zoster ophthalmicus. She was initially treated with steroids and antivirals for an underlying presumed vasculitic cause, but review at 24 hours demonstrated a right central retinal artery occlusion. Embolic screen identified complete occlusion of the right internal carotid artery. She was treated with oral antiviral medication for three weeks but had no visual recovery.

**Conclusion:**

This case highlights an uncommon cause of acute visual loss. We propose that the underlying small and large vessel occlusion in this patient was due to herpes zoster related vasculopathy. A review of the literature is presented to trace the historical perspective of herpes zoster related vasculopathy.

## Introduction

Whilst retinal artery occlusion and stroke are reported in the literature following ipsilateral herpes zoster ophthalmicus, there are few reports of patients having both small and large vessel disease. The earliest reported case of herpes zoster ophthalmicus and central retinal artery occlusion (CRAO) was in 1920 [1]. Since then, there have been eight other reported cases with an onset of CRAO between five days and two years following primary herpes zoster infection [2,3]. Zamora et al reported the case of a 69 year old female who presented with multiple bilateral branch retinal artery occlusions following a bilateral vitritis [4]. This was the first reported case demonstrating the use of polymerase chain reaction analysis of vitreous fluid to diagnose herpes zoster related vasculopathy.

Herpes zoster associated cerebrovascular accident (CVA) has been well documented since 1951 [5]. Gilbert and colleagues proposed in 1974 that the herpes zoster associated CVA was attributable to cerebral angiitis or granulomatous angiitis [6]. The mechanism is likely viral tracking to the trigeminal ganglion and into regional blood vessels such as the internal carotid artery (ICA) via the sympathetic nerves. Biopsy at post mortem following CVA has in some cases identified herpes zoster in the arterial media with and without associated angiitis [7,8].

HZO related vasculopathy remains under-recognised as a mechanism for CRAO.

## Case presentation

A 44-year-old Caucasian woman was referred to the ophthalmology unit four hours after sudden painless loss of vision in her right eye. She was in remission following six months of chemotherapy with cyclophosphomide and fludarabine for chronic lymphocytic leukaemia. Two months after her chemotherapy ceased (9 months prior to this presentation) she had severe right sided herpes zoster ophthalmicus with uveitis that was treated with topical steroids. She was a smoker and on hormone replacement therapy.

Her visual acuity was count fingers at one metre in the right eye and 6/6 in the left eye. Pupils were equal and reactive with a right relative afferent pupillary defect. Dilated fundus examination at presentation was essentially normal showing healthy discs with some mild arteriolar attenuation in the right eye. Whilst the history was highly suggestive of a central retinal artery occlusion (CRAO) optic neuritis could not be excluded. Dual therapy was commenced with ocular massage and intravenous acetazolamide and high dose intravenous (IV) methylprednisolone (1 gram daily) and oral famciclovir (500 mg TDS) for a possible underlying vasculitic cause or optic neuritis. She continued the IV steroids for a total of three days and the oral famciclovir for three weeks.

**Figure 1:**
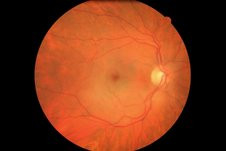
**Colour fundus photograph (right) showing the classic cherry red spot appearance of CRAO**.

Dilated fundus examination of the right eye the following day demonstrated a cherry red spot and retinal oedema, findings consistent with right CRAO (Figure 1). The early and late phase fluorescein angiogram images showed reperfusion of the right central retinal artery with no signs of optic neuritis or retinal vasculitis.

**Figure 2:**
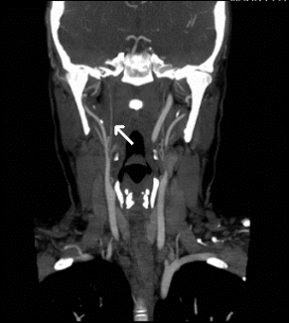
**CT angiogram of carotid vessels with complete occlusion (no contrast) of the right ICA (arrow)**.

An embolic screen for possible sources of emboli included routine blood tests, carotid doppler ultrasound and a transthoracic echocardiogram. Blood tests were normal with no active leukaemia and erythrocyte sedimentation rate of 19 and c-reactive protein < 3. The carotid doppler ultrasound revealed complete occlusion of the right ICA with a patent left ICA. CT angiogram the same day supported the finding of 100% right ICA occlusion with no signs of calcification and demonstrated a generalized diminished right intracranial circulation (Figure 2).

Vascular surgery was not undertaken as the occlusion was total and stable and the right intracranial circulation was well collateralized. At one month follow up she was clinically stable with no visual recovery.

## Conclusion

It is proposed that this patient had a right CRAO due to herpes zoster associated vasculitis of the internal carotid artery and secondary complete arterial occlusion. CRAO as a herpes zoster ophthalmicus related vasculopathy remains under recognised due to the lack of histopathological evidence of causation. Although the pathogenesis of this entity is of interest, the visual outcome after CRAO continues to have a poor prognosis.

## Consent

Written informed consent was obtained from the patient at the time of obtaining photographs for publication of this case report and accompanying images. A copy of the written consent is available for review by the Editor-in-Chief of this journal.

## Competing interests

The authors declare that they have no competing interests.

## Authors' contributions

JC reviewed the case notes, performed a literature review and wrote the manuscript. JB, KK and AH contributed significantly to drafting the manuscript and reviewing for content. All authors were involved in the patient management and read and approved the final manuscript.
